# Current Practice of Assessing and Monitoring Muscle Strength, Muscle Mass and Muscle Function during Nutritional Care by Dietitians in Switzerland—An Online Survey

**DOI:** 10.3390/nu14091741

**Published:** 2022-04-22

**Authors:** Katja Uhlmann, Fabienne Schaller, Undine Lehmann

**Affiliations:** Team Applied Research and Development in Nutrition and Dietetics, Department of Health Professions, Bern University of Applied Sciences, Murtenstrasse 10, 3008 Bern, Switzerland; fabienne.c.schaller@gmail.com (F.S.); undine.lehmann@bfh.ch (U.L.)

**Keywords:** muscle mass, muscle strength, muscle function, dietitian, malnutrition, sarcopenia, survey, nutritional assessment, nutrition-focused physical exam

## Abstract

Muscle parameters are recommended as diagnostic criteria for malnutrition and sarcopenia in various guidelines. However, little is known about the application of muscle parameters in daily practice of nutritional care. The aim of this study was to investigate the current practice of the application of muscle parameters, along with its promoting factors and barriers by dietitians in Switzerland. A 29-item literature-based online survey was developed and distributed via the Swiss professional association of dietitians. The data were analyzed descriptively, and relationships between demographic data and usage were examined. Dietitians (*n* = 117) from all three language regions completed the survey and were included in the analysis. Musculature was classified as important for the assessment of nutritional status. Body weight (89.7%), handgrip strength (87.2%), bioimpedance analysis (BIA) (87.1%) and Body Mass Index (66.7%) were considered as most significant for evaluation of nutritional status. Seventy-point nine percent (70.9%) of dietitians include at least one muscle parameter in their assessment; BIA was the parameter most often included (73.5%). However, the frequency of use of muscle parameter in daily practice was rather low. Only 23.1% applied BIA on a weekly basis. Lack of knowledge (78.6%), practical experience (71.8%) and lack of equipment (77.8%) were most frequently stated as barriers for usage. The general application of muscle parameters in nutritional care is still lacking. There is an opportunity to further strengthen diagnosis and patient monitoring via a stronger application of muscle parameters in daily practice. Practical training and education could help promote their application.

## 1. Introduction

Malnutrition and the risk for malnutrition in patients are found in different settings: hospitals, rehabilitation wards, nursing homes and in the community. It is associated with various adverse outcomes such as increased mortality, longer hospital length of stay, impairments on functionality and quality of life, the risk of institutionalization and higher healthcare cost [1,2,3,4]. Nutritional support in malnourished patients or patients at risk for malnutrition reduced in-hospital mortality and readmission rates [5]. Because malnutrition is characterized by a decrease in fat-free mass, it can be accompanied by sarcopenia [6,7,8,9]. Recent studies show that the prevalence of malnutrition-sarcopenia syndrome in elderly hospitalized patients is about 5%, and it is associated with a higher risk of mortality [7,8]. Therefore, diagnosis and therapy for malnutrition and sarcopenia are of importance and the musculature plays a central role. Furthermore, numerous publications emphasize the importance of the muscle in health and disease [10,11,12,13,14,15].

The European Working Group on Sarcopenia in Older People (EWGSOP) defined a reduced muscle strength and muscle mass as diagnostic criteria for sarcopenia and a reduction in muscle function as criterion to classify the severity of sarcopenia [16]. In addition, the Global Leadership Initiative on Malnutrition (GLIM) considers the presence of reduced muscle mass as an assessment criterion for the diagnosis of malnutrition [17]. Furthermore, muscle parameters such as handgrip strength and gait speed are a predictor for patient outcomes [18,19,20,21,22]. For example, handgrip strength is a prognostic factor for mortality, complications, length of hospital stay and physical functionality [18,19,21,22]. In addition, handgrip strength is often used as an outcome parameter in nutritional studies [23,24]. These points highlight the relevance of muscle parameters for the assessment of nutritional status.

The Nutrition Care Process (NCP) is a process model for dietitians to provide nutritional care and consists of the four steps nutrition assessment, diagnosis, intervention, monitoring and evaluation [25]. Thus far, nutrition assessment is not fully standardized [26]. The nutrition-focused physical examination (NFPE) is part of the nutrition assessment and includes among others the assessment of muscle mass, strength and function, which are important parameters for the evaluation of nutritional status [17,25,27,28,29,30].

Despite the expert guidelines and clinical evidence, little is known about the application of muscle parameters in current dietetic practice so far. There are only a few studies that assess the current practice of muscle parameter in dietetic practice and they indicated a low to moderate application of muscle parameter [31,32,33,34]. Mordarski and Hand found that 48.6% of the surveyed dietitians apply the NFPE, of which 26% use handgrip strength and 37% use functional assessment [33]. A further survey among dietitians in the U.S. suggests that prior training on methods and equipment is a determining factor for applying muscle parameters in practice, because dietitians with prior training in NFPE were significantly more likely to conduct bioelectrical impedance analysis (BIA) than those without training [32]. In Switzerland, published data of the application of muscle parameters in nutritional care are lacking. The application of handgrip strength was investigated in a bachelor thesis of the division Nutrition and Dietetics at the Bern University of Applied Sciences. Only 22% of the surveyed hospitals and private practices applied handgrip strength in their current nutritional care [31].

Further details are required for a better understanding of the current practice and needs of dietitians for using suitable and outcome-driven tools and measurements. The aim of this study was to understand the current clinical practice of dietitians around the assessment of muscle strength, mass, and function in Switzerland for nutrition assessment and patient monitoring.

## 2. Materials and Methods

### 2.1. Study Design

An empirical, quantitative approach was chosen to achieve the study objective. The design was a descriptive study of populations through survey research [35].

### 2.2. Development of an Online Questionnaire

**Figure 1:**
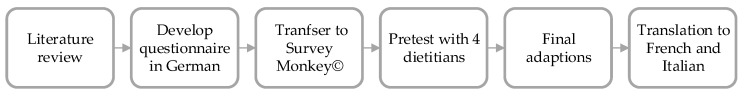
Procedure for developing the online questionnaire.

The development of the questionnaire is illustrated in Figure 1. A first version of the online questionnaire was developed based on a literature review and was transferred in the tool SurveyMonkey© (1999–2021). The questions were derived from a conducted unpublished Bachelor thesis from the division of Nutrition and Dietetics of the Bern University of Applied Sciences and already published studies on this issue [32,33,36,37,38,39]. A pretest was conducted to review the comprehensibility and the structure of the questionnaire. The pretest was conducted by four dietitians with experience in dietetic practice and survey development from the division of Nutrition and Dietetics at the Bern University of Applied Sciences. Based on the responses from the pretest a final adaptation of the online questionnaire was made. Main adaptations concerned the reduction in length and complexity by reducing the number of matrix questions and number of items within the matrix questions. Furthermore, wording, spelling mistakes and technical errors in the online survey were corrected, and an introduction was added to guide the participants through the questionnaire and explained abbreviations and tests. Following these adaptations, the questionnaire was translated into French and Italian and finalized as a multilingual version in SurveyMonkey© (1999–2021).

Based on the gap in literature, the focus of the questionnaire was set on the current practice of usage of muscle parameters and potential enablers and barriers. Furthermore, demographic data of the participants were assessed within the survey. The length of the survey was also considered when compiling different items. The questionnaire consisted of 29-items and included questions on the following aspects: one question related to inclusion/exclusion criteria, eight questions about perception and attitudes towards muscle parameters, seven questions about application, two questions about promoting factors and barriers for application, seven demographic questions, three questions about acquisition of knowledge and a general comment. The following parameters were defined as muscle parameters: BIA, Magnetic Resonance Imaging (MRI), computed tomography (CT), Dual-Energy-X-Ray-Absorptiometry (DXA) as muscle mass, handgrip strength as muscle strength, Timed Up and Go (TUG), Short Physical Performance Battery (SPPB), chair-stand test and 400 m walking test as muscle function according to the diagnostic criteria for sarcopenia [16]. Body weight, Body Mass Index (BMI) and upper arm/calf circumference were referred to as parameters in the context of the questionnaire but not as muscle parameters. Different types of questions were used. Multiple choice questions either with one or more answer options as well as 5-point-likert scales. Additionally, matrix questions, rating questions (scale 0% not at all relevant to 100% highly relevant) and open response fields were used. The translated questionnaire can be found in Supplementary Materials (SQ1).

### 2.3. Distribution of the Questionnaire

The online questionnaire was distributed over the mailing list of the Swiss Association of Dietitians (SVDE)—the professional association of dietitians in Switzerland. This approach allowed reaching dietitians from German, French and Italian language regions and from different fields of activity (working in hospitals, rehabilitation centers, other institutions, private practice or the community setting). The mailing list included active members of the SVDE and students of nutrition and dietetics. Honorary members and pensioners were excluded from the mailing list. In addition, team members of the division of Nutrition and Dietetics from the Bern University of Applied Science distributed the survey via their professional network, more specifically via LinkedIn profiles, among the master’s students of the study program Master of Science in Nutrition and Dietetics and during the conference Nutrition 2021 from 24 to 26 June 2021. To include only dietitians with patient contact, a first triage question was included. Respondents that answered that they had no patient contact were forwarded automatically to the end of the survey and were excluded. The survey was open for four weeks in June 2021. After two weeks, a reminder was sent via the SVDE and LinkedIn. Respondents were not compensated for participation in this study.

### 2.4. Data Analysis

Survey results were downloaded from SurveyMonkey© (1999–2021) and checked for completeness, missing values and obvious errors. Only fully completed questionnaires were included in the analyses to relate them to the demographic responses at the end of the questionnaire. Abandoned questionnaires were excluded from further analysis.

The data were analyzed descriptively using the statistic software SPSS version 27 (IBM, Armonk, NY, USA) and Microsoft Excel, Version 2008 (© Microsoft, Redmont, WA, USA). Furthermore, analyses of relationships between different demographic variables and the application of muscle parameters/integration into nutrition assessment via contingency tables with Fisher’s exact test and contingency coefficient were conducted. Language region, education, professional experience and professional environment were the demographic data that were used for the analysis of relationships.

## 3. Results

Respondents numbering 177 started the survey, of which 9 were excluded, because they declared to have no direct patient contact and were, therefore, not part of the target group. Respondents numbering 117 finalized the survey with a complete set of responses and were included in the results, while 51 respondents were excluded because of missing data.

### 3.1. Demographic Data of the Respondents

The demographic data of the respondents is shown in Table 1. The majority of respondents had a Bachelor of Science (75.2%) as their highest education, worked in a hospital (44.4% mainly with inpatients resp. 13.7% mainly with outpatients) and had a professional focus on malnutrition (83.8%, multiple answers possible). The mean age of the respondents was 39.3 (SD 11.2) years, and they had on average 13.6 (SD 10.9) years of professional experience as a dietitian. Two respondents did not provide information about their age. Seventy-one point eight percent (71.8%) of the respondents were from the German-speaking region, followed by 24.8% from French-speaking and 3.4% Italian-speaking regions. Most respondents worked in a hospital (58.1%). Of these (*n* = 68), the majority worked in a cantonal hospital (48.5%), followed by regional hospital (33.8%), private clinic (10.3%) or a university hospital (7.4%).

### 3.2. Perception and Attitudes towards Muscle Parameters

All respondents (*n* = 117, 100%) agreed with the statement that musculature is important for the assessment of nutritional status. The relevance of muscle parameters (muscle mass, strength and function) for their nutritional care was scaled at 75.0% (median) from a scale of 0% (not at all relevant) to 100% (highly relevant). When asked about the muscle parameter with the greatest significance for nutritional care, the respondents chose muscle mass (51.3%) followed by muscle strength (31.6%) and muscle function (17.1%). When a selection of single parameters was given, body weight was the measurement rated of highest importance for the assessment of nutritional status if taking the two response options “very important” and “rather important” together (89.7%). This was followed by handgrip strength (87.2%), BIA (87.1%), BMI (66.7%) and upper arm/calf circumference (63.2%) (data are shown in the Supplementary Materials, Table S1). The following statements about the benefits of using muscle parameters received the greatest agreement from respondents: “Provides objective data” (90.6%), “Increases the added value of nutritional care” (80.3%), “Adds new scientific evidence to daily clinical practice” (78.6%) and “Has a positive effect on interprofessional collaboration” (73.5%) (Supplementary Materials, Table S2).

In Table 2 and Table 3, the results on the questions “who should perform the measurement of the different parameters” and “who should interpret them” are presented (multiple answers possible). There is a very high agreement that BIA should be measured (94.0%) and interpreted (97.4%) by dietitians. Handgrip strength should be measured both by dietitians (71.8%) and physiotherapists (70.1%). The assessments of functional tests such as TUG and walking tests were seen rather in the role of physiotherapists than in the role of dietitians. However, interpretation was with about 32–39% within the scope of dietitians. Dual-Energy X-ray Absorptiometry, MRI and CT should be measured and interpreted mainly by doctors (76.9% and 87.2%, respectively) according to respondents.

### 3.3. Application of Muscle Parameters

Of the respondents, 70.9% reported to have included at least one muscle parameter in their nutritional assessment, and 74.4% included at least one muscle parameter in their monitoring. Bioimpedance analysis was most frequently applied for nutrition assessment (73.5%, multiple answers were possible) as well as for monitoring (85.1%), followed by handgrip strength (49.4% resp. 50.6%) (data are presented in the Supplementary Material, Table S3). Bioimpedance analysis (82.9%) and handgrip strength (64.1%) were also considered as parameters with the highest benefit for monitoring, followed by functional parameters such as walking test (25.6%) or TUG, chair-stand test or Short Physical Performance Battery (SPPB) (23.9%) (Table S3).

**Figure 2:**
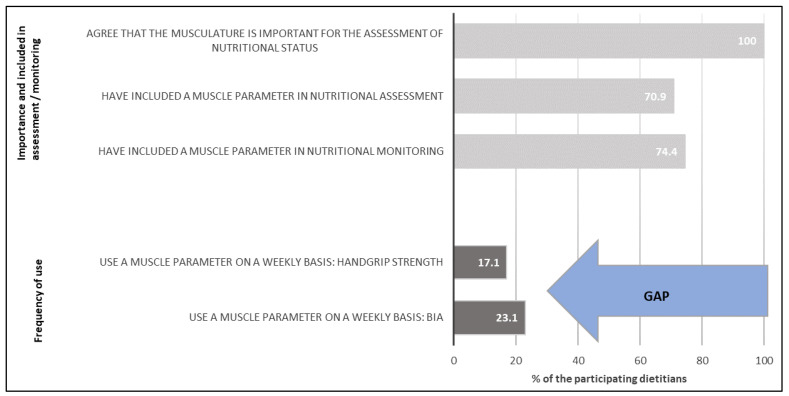
Perceived importance of the musculature, inclusion of muscle parameters for nutritional assessment and monitoring and application of the most common muscle parameters handgrip strength and BIA by the participating dietitians. Note the gap between the knowledge about the importance of musculature and the daily use of the muscle parameters (blue arrow).

Table 4 shows the frequency of application of single muscle parameters in dietary practice. While body weight was applied by 93.2% of respondents on a weekly basis, BIA was applied only by 23.1% and handgrip strength was applied by 17.1%. What is worth mentioning is that handgrip strength is never applied by 41.9% of respondents. Upper arm/calf circumference was never applied by more than half (59.8%) of the respondents and DXA, MRI or CT were applied less than 5 times per year by 90.6% of respondents. In addition to body weight and BMI, all other parameters showed a wide variation, from never applied to very often applied, as shown in Table 4. Figure 2 summarizes the results on perceived importance and application of muscle parameters.

Respondents were asked which profession performs the measurements of the different muscle parameter at the current workplace (Table 5). Most dietitians measured BMI (76.9%) and BIA (71.8%). Handgrip strength was measured by 35% of dietitians. Upper arm/calf circumference was stated in the majority as not measured by any profession. The functional muscle parameters are mainly conducted by physiotherapists (58.1% TUG, Chair Stand Test or SPPB resp. 60.7% walking test).

Regarding promoting factors for the measurement and interpretation of muscle parameters, it was found that the statements “Good knowledge/understanding of the measurement methods/parameters” (92.3%) and “Sufficient practical training/application experience” (87.2%) as well as “Availability of the device” (83.8%) were considered the most important, followed by “Sufficient scientific evidence (e.g., available reference values/knowledge of the state of research)” (75.2%) and “Sufficient time for application” (72.6%). Barriers are considered mostly the opposites such as “Lack of knowledge/understanding of the methods/parameters” (78.6%), “Lack of devices” (77.8%) and “Lack of practical training/application experience” (71.8%) 

### 3.4. Self-Estimation Regarding Knowledge

In Table 6, the estimation of self-knowledge on the different parameters is shown. Most respondents thought they had a very good knowledge about BMI (77.8%) and body weight (76.9%). Bioimpedance analysis and handgrip strength were estimated as very good and rather good by 74.4% and 56.4% of dietitians, respectively. Dietitians considered their knowledge about DXA, MRI and CT as well as the functional tests in the majority as poor and rather poor. Ninety-two point three percent (92.3%) of respondents were interested in acquiring more information/practical skills about muscle parameters.

### 3.5. Relationships between Demographic Data and the Application of Muscle Parameters

There was a significant association between the language region and the application of muscle parameters for monitoring, indicating that less French-speaking dietitians apply muscle parameters for monitoring compared to German and Italian-speaking dietitians. However, the strength of association was small (contingency coefficient 0.246; *p* < 0.05 see Supplementary Material Table S4). No associations were found between education level and the following variables: application of muscle parameters for monitoring (*p* = 0.513), inclusion of muscle parameters in the nutrition assessment (*p* = 0.866) or which of the three parameters, muscle mass, strength and function, have the highest significance for nutritional care (*p* = 0.637). There was also no association between the professional environment and whether at least one muscle parameter was included in the nutrition assessment (*p* = 0.812). There was no association between professional experience and the integration of a muscle parameter in nutrition assessment (*p* = 0.627).

## 4. Discussion

Our study adds important information to the international context, where data about this subject are limited. The muscle has structural and metabolic functions, and muscle atrophy related to age and disease led to adverse health and economic consequences [10,40]. Therefore, integrating muscle mass assessment in the daily practice of dieticians and health professionals is of importance.

All respondents (*n* = 117) considered that musculature is important for the assessment of nutritional status. Most respondents rated all parameters, except DXA, MRI or CT (45.3%), as very important or rather important for assessing nutritional status. Looking at the frequency of application, an obvious gap between application and perception of importance becomes visible. Most dietitians had a muscle parameter included in the assessment or monitoring in their daily practice. Even though BIA and Handgrip strength were mentioned most often to be included in assessment and monitoring (BIA 73.5% resp. 85.1%; handgrip strength 49.4% resp. 50.6%), they were applied on a weekly basis by only 23.1% (BIA) and 17.1% (handgrip). Similar results about application of muscle parameters were also observed in other studies. Girsemihl and colleagues investigated variables in nutrition assessment in German dietitians and nutritionists with clinical practice through a 3-item Likert-scale online survey [41]. While weight and height were assessed regularly by 100% of the dietitians, BIA was applied only by 17% of dietitians regularly and 31% if necessary, upper arm circumference was applied by 12% regularly and 32% if necessary, and handgrip strength was applied by 7% regularly and 24% if necessary [41]. Similar findings in the frequency of applying parameters >1 x per week (very often) were observed in this study as well for body weight (93%) and BIA (23%), but a higher application of handgrip strength and lower application of upper arm/calf circumference (>1 x per week, 17% resp. 3%) was found. Furthermore, BMI was used by 86.3% very often (>1 x per week). One possible reason for the frequent use of body weight and BMI in dietetic practice could be, among being cheap, quick and simple to use, that both were defined as one component to estimate the malnutrition risk. Screening tools for malnutrition such as Nutritional Risk Screening (NRS 2002), Malnutrition Universal Screening Tool (MUST) or Mini Nutritional Assessment (MNA) all include thresholds for BMI and unintentional loss of body weight [42]. However, in our survey, the use of different parameters for nutritional assessment and monitoring in general was assessed but not details about the specific purposes in relation to individual parameters. The data obtained from this study are also comparable with previous results about the application of handgrip strength as a muscle strength parameter from the US (26.1%), Australia (22.7%) and Switzerland (22%) [31,33,34]. There are no previous data on the application of BIA, MRI, DXA or CT by dietitians in Switzerland. Results on functional tests of muscle function such as SPPB, TUG, chair-stand and 6-minute walk tests are rare, with no results in Swiss clinical practice.

Dietitians are the profession that most often performed the measurement of BIA (71.8%) and BMI (76.9%) at their current workplace compared to other professions, while upper arm/calf circumference was in the majority assessed by nobody (65.8%). Respondents were asked in the present study which profession should perform measurements and interpret muscle parameters. While dietitians understand that their own role is to perform and interpret BIA (94.0% resp. 97.4%), BMI (76.1% resp. 97.4) and handgrip strength (71.8% resp. 84.6%), they considered that functional tests such as TUG and walking test should rather be performed and interpreted by physiotherapists (TUG 92.3% resp. 91.5% and walking test 95.7% and 94.9%); and DXA, CT and MRI by doctors (76.9% resp. 87.2%). Only 17.1% of dietitians considered that they should conduct a TUG, chair-stand or SPPB tests, while a slightly higher number of dietitians considered that they should be able to interpret these tests (39.3%). The results suggest that conducting functional measurements is not part of dietitians’ understanding of their role, but to some extent, interpreting measurements already available is part of their role. Understanding the clinical role of dietitians in Switzerland can also partly explain the mentioned gap between the perception of importance of muscle parameters and current application. For the question which profession performs the measurement of the parameters at the current workplace, there was a higher number of participants that chose “no responses” as an answer compared to the other questions. This could indicate greater uncertainty relative to which professionals measure which muscle parameters.

Although the results indicate a rather low application of muscle parameters in nutritional care, in our investigation, respondents see advantages in the application. Most frequently, they agreed with the following statements regarding the benefits of application: “Provision of objective data”, “Increased added value of nutritional care”, “New scientific knowledge for daily clinical practice” and “Positive impact on interprofessional collaboration”. The benefit for interprofessional collaboration has been mentioned elsewhere, concluding that the application of NFPE would highlight the importance of dietitians in an interdisciplinary team [43]. Furthermore, the inclusion of NFPE in education and training would increase the value of dietitians in diagnosing and treating nutrition-related problems [30]. This would promote the acceptance of physical assessment skills as a standard of practice for dietitians.

Several barriers for the application of muscle parameters can be highlighted. Similarly to this study, a lack of knowledge and practical experience [32,33,38], lack of devices [38,41] and time requirement [33,36,38] were mentioned in the literature. The self-assessment of knowledge and the frequency of application of individual parameters coincides. Self-assessment of knowledge about BMI (77.8%) and body weight (76.9%) was high, and correspondingly, the frequency of application of these two parameters was also high. Ninety-three point two percent (93.2%) of respondents apply body weight on a weekly basis and 86.3% apply BMI more than once a week. In contrast, most dietitians considered their knowledge about DXA, MRI and CT as well as the functional tests as poor and rather poor. Low values were also shown in the application of these parameters. DXA, MRI and CT were never applied (<1 x/year) by 80.3% of the respondents, walking tests were not applied by 65.0% and TUG, Chair Stand Test or SPPB were not applied by 68.4%. However, it is not possible to make a more precise distinction between whether the lack of knowledge is a barrier to application or whether the low knowledge is due to low applications. Furthermore, low application and knowledge could be related to professional role understanding. Training as an important enabler of usage of muscle parameters was confirmed in previous studies. Additional NFPE training resulted in a significantly higher number of NFPE components applied by dietitians [44]. A study by Reijnierse and colleagues recorded the application of parameters and measurements in addition to a change in knowledge about sarcopenia [38]. Before training, 23.5% of dietitians measured muscle mass (using BIA), and 29.4% measured handgrip strength, while 0% measured walking speed (4 m walk test). After training, 88.2% had the intention to collect muscle mass. For handgrip strength, the intention was 70.6% and for walking speed, it was 23.5%. These data show the high importance of specific hands-on training for the application of muscle parameters and were also confirmed by further studies [32,44].

The lack of time, corresponding to the difficulty of taking in charge the costs for the measurements, was identified as one of the barriers in the current investigation. Deutz et al. concluded in their review that the economic benefits of strengthening the application of muscle parameters, specifically muscle mass for malnutrition, need to be better communicated to payers and those in charge of funding decisions [45]. This demand can be supported based on our results.

Only very limited data are available on dietitians’ perceptions of muscle strength, mass and function as parameters for nutrition assessment or monitoring generally and especially in German-speaking countries. Further research is also needed on the application of individual parameters. For example, it would be interesting to investigate why functional tests that do not require devices, such as the TUG or the 6-minute walk test, are performed less often than the assessment of muscle parameters, which require a device such as handgrip strength or BIA. In particular, the finding was that device availability is a promotor, respectively, and the lack of devices is a barrier. The lack of knowledge about the performance of these tests and consideration of these tests is beyond the scope of the role of a dietitian, and this could be a possible reason why functional tests are not used more often. Future work should address these research questions for a deeper understanding of the promoting factors and barriers for usage with a qualitative research design. Furthermore, investigating which diseases and therapeutic interventions for monitoring muscle parameters are already included in the standard of care and those that have good potential based on available scientific evidence is of interest. This question would need to be answered specific settings depending on the workplace, i.e., acute hospital versus rehabilitation clinic or ambulatory practice. Additionally, a better understanding of the perceived scope of work and interprofessional collaboration, e.g., with the physiotherapy could further help understand the gap in the usage of muscle parameters. Finally, the question whether a standardized assessment including muscle parameters improves clinical outcomes should be answered, for example, within the framework of outcomes research.

**Figure 3:**
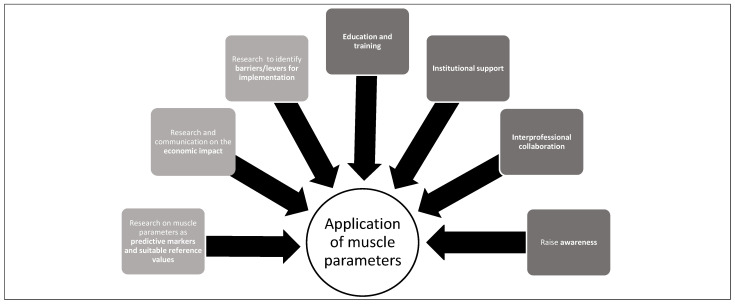
Requirements that could strengthen the application of muscle parameters in daily practice.

In the summary in Figure 3, our study, also supported by other authors [38,45], highlights the following needs in order to strengthen the application of muscle parameters in daily dietetic practice:

- Raise awareness among healthcare professionals concerning the application of muscle parameters (muscle mass, strength and function) to assess and monitor malnutrition and sarcopenia and initiating appropriate nutritional care to improve patient outcomes;
- Develop and encourage attendance in training on methods and tools for assessing muscle mass, strength and function;
- Strengthen interprofessional collaboration for patient screening, assessment and management among health professionals;
- Institutional support to provide the necessary conditions to promote the application of muscle parameters in daily practice;
- Research and communication on the economic impact of the inclusion of muscle parameters in the scope of practice;
- Research to identify barriers for implementation and steps to overcome these barriers, as well as specific research on muscle parameters as predictive markers and suitable reference values.

### Strengths and Limitations

A strength of the survey was the wide distribution in all three language regions in Switzerland via SVDE so that more than 1400 people were contacted. A comprehensive view on the applications of different muscle parameters, perception and attitudes; knowledge; promoting factors; and barriers of usage could be gained for the first time in Switzerland.

Since the questionnaire was shared via the professional network, an exact response rate cannot be calculated. The response rate of completed questionnaires based on the SVDE distribution was 8% in a comparable range relative to response rates of 6–9% in professional surveys conducted with dietitians [33].

Due to the drop out of respondents and the rather low response rate, a certain bias of respondents can be expected. The respondents might have been interested in the topic and might have had a better perception and higher application of muscle parameters. The average professional experience of 10 years showed that respondents were on average experienced professionals. These results might, therefore, not completely reflect the current practice in Switzerland.

Another limitation was that the questionnaire was not validated but only tested for comprehensibility and content through the pretest by expert dietitians. There were no tests of validity or reliability. The authors are nevertheless confident that the findings reflect the current situation in Switzerland well, based partly on the literature and expert opinions.

## 5. Conclusions

For the first time, a dedicated survey delivered a comprehensive view on the application of various muscle parameters in dietary practices in Switzerland. While there is a profound knowledge of the importance of muscle parameters for the assessment of the nutritional status and for patient monitoring, there is still a gap in the actual application of muscle parameters for assessment and monitoring in nutritional care. A successful inclusion of muscle measurements in clinical practice provides opportunities to improve screening and assessment and can evaluate the progress and efficacy of nutrition interventions that impact patient outcomes and strengthen dietetic practice. Factors were identified to improve the application of muscle parameters. Training that includes practical exercise could be one enabling factor to close the gap between perception and application. Further qualitative research is necessary to better understand the promoting factors and barriers for the application of muscle parameters in nutritional care in order to overcome current hurdles and to promote more general applications.

## Figures and Tables

**Table 1:** Demographic data of the respondents: education, professional environment and professional focus (*n* = 117).

Demographic Data of Respondents	*n*	%
**Highest education**		
Student (in education)	2	1.7
College of higher education	12	10.3
Bachelor of Science	88	75.2
Master of Science	14	12.0
PhD	1	0.9
**Professional environment**		
Private practice/freelance	25	21.4
Hospital (mainly inpatient)	52	44.4
Hospital (mainly outpatient)	16	13.7
Rehabilitation clinic	16	13.7
Nursing home	3	2.6
Other	5	4.3
**Professional focus ^1^**		
Malnutrition	98	83.8
Adiposity	74	63.2
Metabolic diseases	62	53.0
Cardiovascular disease	43	36.8
Diseases of the digestive system	58	49.6
Kidney diseases	23	19.7
Allergies/intolerances	24	20.5
Other	15	12.8

^1^ multiple answers possible.

**Table 2:** Response frequencies to the question on who should perform the measurement of the different parameters (*n* = 117, multiple answers possible).

Parameter	Response Frequency %							
	Dietitians	Doctors	Nurses	Physio-Therapists	Other Therapists	Nobody	Not Important	No Answer
Body weight	59.0	30.8	78.6	7.7	3.4	0.0	0.9	0.0
BMI	76.1	36.8	52.1	6.0	3.4	0.0	5.1	0.0
Upper arm/calf circumference	58.1	20.5	29.1	37.6	2.6	0.9	1.7	6.8
BIA	94.0	10.3	6.8	11.1	6.0	0.0	0.9	1.7
DXA, MRI, CT	11.1	76.9	10.3	3.4	9.4	1.7	2.6	7.7
Handgrip strength	71.8	15.4	18.8	70.1	19.7	0.0	0.9	0.9
TUG, Chair Stand Test or SPPB	17.1	8.5	14.5	92.3	6.0	0.0	0.0	4.3
Walking test	11.1	8.5	12.0	95.7	6.8	0.0	0.0	1.7

Abbreviations: Body Mass Index (BMI), bioimpedance analysis (BIA), Dual-Energy-X-ray-Absorptiometry (DXA), Magnetic Resonance Imaging (MRI), computed tomography (CT), Timed Up and Go (TUG) and Short Physical Performance Battery (SPPB).

**Table 3:** Response frequencies to the question on who should interpret the different parameters (*n* = 117, multiple answers possible).

Parameter	Response Frequency %							
	Dietitians	Doctors	Nurses	Physio-Therapists	Other Therapists	Nobody	Not Important	No Answer
Body weight	98.3	72.6	37.6	13.7	2.6	0.0	0.0	0.0
BMI	97.4	65.8	27.4	11.1	2.6	0.0	0.9	0.0
Upper arm/calf circumference	71.8	44.4	11.1	46.2	0.9	0.0	1.7	6.8
BIA	97.4	47.0	3.4	17.9	0.9	0.0	0.0	0.9
DXA, MRI, CT	32.5	87.2	6.0	7.7	3.4	0.0	0.9	7.7
Handgrip strength	84.6	43.6	14.5	72.6	19.7	0.0	0.0	0.9
TUG, Chair Stand Test or SPPB	39.3	45.3	15.4	91.5	9.4	0.0	0.0	4.3
Walking test	32.5	46.2	14.5	94.9	9.4	0.0	0.0	2.6

**Table 4:** Frequency of application of muscle parameter in dietary practice (*n* = 117).

Parameter	Response Frequency %					
	Never (<1 x/Year)	Rare (<5 x/Year)	Occasionally (<10 x/Year)	Frequently (>1 x/Month)	Very Often (>1 x/Week)	No Answer
Body weight	0.0	0.0	0.0	6.8	93.2	0.0
BMI	0.0	0.9	3.4	9.4	86.3	0.0
Upper arm/calf circumference	59.8	19.7	9.4	6.0	3.4	1.7
BIA	17.9	13.7	17.1	28.2	23.1	0.0
DXA, MRI, CT	80.3	10.3	3.4	0.9	0.0	5.1
Handgrip strength	41.9	16.2	8.5	16.2	17.1	0.0
TUG, Chair Stand Test or SPPB	68.4	10.3	8.5	6.8	2.6	3.4
Walking test	65.0	11.1	7.7	9.4	3.4	3.4

**Table 5:** Response to the question who performs the measurement of the single parameters at the current workplace (*n* = 117, multiple answers possible).

Parameter	Response Frequency %							
	Dietitians	Doctor	Nurse	Physio-Therapists	Other Therapists	Nobody	Not Important	No Answer
Body weight	51.3	24.8	74.4	0.9	2.6	1.7	0.9	0.9
BMI	76.9	33.3	37.6	0.9	1.7	2.6	1.7	1.7
Upper arm/calf circumference	17.9	3.4	4.3	5.1	0.0	65.8	0.9	9.4
BIA	71.8	6.0	4.3	2.6	5.1	16.2	1.7	4.3
DXA, MRI, CT	0.9	34.2	3.4	0.9	3.4	46.2	1.7	14.5
Handgrip strength	35.0	2.6	1.7	22.2	21.4	32.5	1.7	7.7
TUG, Chair Stand Test or SPPB	4.3	0.9	2.6	58.1	0.0	25.6	0.9	11.1
Walking test	2.6	0.9	1.7	60.7	0.9	26.5	0.9	8.5

**Table 6:** Self-Estimation regarding knowledge on single parameters (*n* = 117).

Parameter	Response Frequency %					
	Very Good Knowledge	Rather Good Knowledge	Neither Good/Nor Bad Knowledge	Rather Poor Knowledge	Poor Knowledge	No Answer
Body weight	76.9	21.4	1.7	0.0	0.0	0.0
BMI	77.8	21.4	0.9	0.0	0.0	0.0
Upper arm/calf circumference	6.0	21.4	28.2	33.3	10.3	0.9
BIA	29.1	45.3	13.7	8.5	2.6	0.9
DXA, MRI, CT	0.0	9.4	21.4	26.5	39.3	3.4
Handgrip strength	13.7	42.7	15.4	20.5	7.7	0.0
TUG, Chair Stand Test or SPPB	3.4	21.4	20.5	24.8	28.2	1.7
Walking test	3.4	23.9	21.4	22.2	27.4	1.7

## Data Availability

The data presented in this study are available on request from the corresponding author. The data are not publicly available due to privacy reasons. Although the data were collected anonymously, conclusions about individuals could not be completely ruled out due to some specific data on language region, professional environment and highest education in the professional population.

## Supplementary Materials

The following supporting information can be downloaded at: https://www.mdpi.com/article/10.3390/nu14091741/s1, SQ1: Translated questionnaire, Table S1: Importance of single parameters, Table S2: Statements on the use of muscle parameters, Table S3: Inclusion of specific muscle parameters in assessment and monitoring, Table S4: Cross-table language region versus use of muscle parameters for monitoring.
