# Supplementary material for: Current Practice of Assessing and Monitoring Muscle Strength, Muscle Mass and Muscle Function during Nutritional Care by Dietitians in Switzerland—An Online Survey
Source: Nutrients. 2022 Apr 22;14(9):1741. doi: 10.3390/nu14091741 (PMC9103861; doi:10.3390/nu14091741)
Supplement: Supplementary file 1 [file nutrients-14-01741-s001.zip › SQ1.pdf]

Supplementary Materials: **SQ1:** Online questionnaire

Entry question

This question is used to evaluate whether you belong to the target group for this questionnaire.

1. Are you a dietitian working with patients?
Please relate the following questions to your major patient contact activity.

[only one response option]

- ○ Yes go to question 2
- ○ No go to closing for NON - participants

Perception, self-assessment of knowledge and attitude

Now we are interested in your opinions on various aspects related to muscle parameters. First, we would like to know what is your perception, self-assessment of knowledge and attitude towards muscle parameters. We understand muscle parameters as muscle mass, muscle strength and muscle function.

Perception

2. The musculature is important for assessment of nutritional status.

[only one response option]

- ○ I agree go to question 3
- ○ I don't agree go to question 4

3. How relevant are muscle parameters (muscle mass, strength, function) for nutritional care?
0 % = not relevant, 100 % = highly relevant

[Scale from 0 – 100]

4. Which of the three muscle parameters (muscle mass, strength, and function) has the greatest significance for nutritional care?

Explanations of the parameters are at the end of this page.

[only one response option]

- ○ Muscle mass (e.g. BIA, MRI, CT, DXA)
- ○ Muscle strength (e.g. handgrip strength)
- ○ Muscle function (e.g. functional tests like "Timed up and go"-Test, SPPB, Chair - Stand - Test, 400 m – walking test)

Explanations:

BIA: Bioimpedance analysis. Applied for body composition analysis, respectively. measurement of muscle mass.

MRI: Magnetic resonance imaging. Imaging procedure to show the structure and function of tissues and organs in the body. Applied to measure muscle mass.

CT: Computed tomography. Imaging procedure in radiology. Applied to measure muscle mass.

DXA: Dual - X-ray - Absorptiometry. X-ray diagnostic method for determining body composition. Used to measure muscle mass.

Handgrip strength: Applied to measure muscle strength.

«Timed up and go» - test: Simple mobility test for assessing mobility and body balance, as well as estimating the risk of falling. Applied to measure muscle function.

Short Physical Performance Battery: Short SPPB. Test procedure for measuring balance, walking speed, leg strength. Applied to measure muscle function.

Chair – Stand – Test: Measures leg strength and endurance. Applied to measure muscle function.

400 m – walking test: Applied to measure muscle function.

5. How do you rate the importance of each measurement in the assessment of nutritional status?

Explanations of the parameters are at the end of this page.

[only one response option for each parameter]

	Not important at all	Rather not important	Neither	Rather important	Very important	No answer
Body weight						
Body Mass Index						
Upper arm / calf circumference						
BIA						
MRI, CT or DXA						
Handgrip strength						
"Timed up and go" – Test, Chair – Stand – Test or SPPB						
Walking test (e.g. walking speed or 400 m –walking test)						

6. Which statements regarding the use of muscle parameters (muscle mass, strength, function) in nutritional care do you agree with?

Mark all answers that apply.

[multiple response options]

- ☐ Improves the relationship between patient and dietician
- ☐ Worsens the quality of counselling through an additional time-consuming assessment
- ☐ Leads to demotivation in patients when values get worse
- ☐ Adds new scientific evidence to daily clinical practice
- ☐ Provides objective data
- ☐ Leads to a strong focus on objective parameters
- ☐ Increases the added value of nutritional care
- ☐ Increases professional self-esteem
- ☐ Has a positive effect on interprofessional collaboration
- ☐ Worsens interprofessional collaboration if responsibilities are not clarified
- ☐ Others: ______________________________________

<u>Self-assessment of knowledge</u>

7. How would you rate your knowledge on the parameters below?

[only one response option for each parameter]

	Poor knowledge	Rather poor knowledge	Neither good / nor bad knowledge	Rather good knowledge	Very good knowledge	No answer
Body weight						
Body Mass Index						
Upper arm / calf circumference						
BIA						
MRI, CT or DXA						
Handgrip strength						
"Timed up and go" – Test, Chair – Stand – Test or SPPB						
Walking test (e.g. walking speed or 400 m –walking test)						

<u>Attitude</u>

8. In your opinion, who should perform the measurements of the following parameters?

Please answer the question for your main professional environment (e.g. inpatient in hospital or outpatient in hospital). Place a mark each applicable answer.

[multiple response options for each parameter]

	Dietitians	Doctors	Nurses	Physio-therapists	Other therapists	Nobody	Not important	No answer
Body weight								
Body Mass Index								
Upper arm / calf circumference								
BIA								
MRI, CT or DXA								
Handgrip strength								
"Timed up and go" – Test, Chair – Stand – Test or SPPB								
Walking test (e.g. walking speed or 400 m –walking test)								

9. In your opinion, who should interpret the following parameters?

Please answer the question for your main professional environment (e.g. inpatient in hospital or outpatient in hospital).

Mark each applicable answer.

[multiple response options for each parameter]

	Dietitians	Doctors	Nurses	Physio-therapists	Other therapists	Nobody	Not important	No answer
Body weight								
Body Mass Index								
Upper arm / calf circumference								
BIA								
MRI, CT or DXA								
Handgrip strength								
"Timed up and go" – Test, Chair – Stand – Test or SPPB								
Walking test (e.g. walking speed or 400 m –walking test)								

Application

In this section you will be asked questions regarding the practical application of the muscle parameters.

10. Have you integrated at least one muscle parameter (muscle mass, muscle strength and/or muscle function) into your nutritional assessment?

[only one response option]

- ○ Yes go to question 11
- ○ No go to question 12

11. Which muscle parameters have you integrated into your assessment?

[multiple response option]

- ☐ BIA
- ☐ MRI, CT or DXA
- ☐ Handgrip strength
- ☐ «Timed up and go» – Test, Chair - Stand – Test or SPPB
- ☐ Walking test (e.g. walking speed or 400 m –walking test)
- ☐ Others: ______________________________

12. Who principally performs the measurements of the following parameters at your workplace?
Please answer the question for your main professional environment (e.g. inpatient in hospital or outpatient in hospital). Mark each applicable answer.

[multiple response options for each parameter]

	Dietitians	Doctors	Nurses	Physio-therapists	Other therapists	Nobody	Not important	No answer
Body weight								
Body Mass Index								
Upper arm / calf circumference								
BIA								
MRI, CT or DXA								
Handgrip strength								
"Timed up and go" – Test, Chair – Stand – Test or SPPB								
Walking test (e.g. walking speed or 400 m –walking test)								

13. How often do you, as a dietitian, apply the following parameters in your work with patients?

[only one response option for each parameter]

	Never (< 1 x / year)	Rare (< 5 x / year)	Occasionally (< 10 x / year)	Frequently (> 1 x / month)	Very often (> 1 x / week)	No answer
Body weight						
Body Mass Index						
Upper arm / calf circumference						
BIA						
MRI, CT or DXA						
Handgrip strength						
"Timed up and go" – Test, Chair – Stand – Test or SPPB						
Walking test (e.g. walking speed or 400 m –walking test)						

14. Do you apply muscle parameters (muscle mass, strength, function) as a follow-up for nutritional care?

[only one response option]

- ○ Yes go to question 15
- ○ No go to question 16

15. Which muscle parameters (muscle mass, strength, function) do you integrate as a follow-up for nutritional care?

[multiple response options]

- ☐ BIA
- ☐ MRI, CT or DXA
- ☐ Handgrip strength
- ☐ «Timed up and go» – Test, Chair - Stand – Test or SPPB
- ☐ Walking test (e.g. walking speed or 400 m –walking test)
- ☐ Others: ______________________________

16 Which muscle parameters (muscle mass, strength, function) do you find most useful for patient monitoring?

[multiple response options]

- ☐ BIA
- ☐ MRI, CT or DXA
- ☐ Handgrip strength
- ☐ «Timed up and go» – Test, Chair - Stand – Test or SPPB
- ☐ Walking test (e.g. walking speed or 400 m –walking test)
- ☐ Others: ______________________________

Promoting factors / Barriers
In this section, we ask about promoting and inhibiting factors for the application of muscle parameters.

17. Which of the following promoting factors are relevant to you in relation to the measurement and interpretation of muscle parameters (muscle mass, strength, function)?

Mark all answers that apply.

[multiple response options]

- ☐ Good knowledge / understanding of the measurement methods / parameters
- ☐ Sufficient practical training / application experience
- ☐ Defined areas of responsibility interprofessional
- ☐ Willingness of the professional for physical examinations (touch)
- ☐ Good organisation / scheduling
- ☐ Available location / room
- ☐ Sufficient time for application
- ☐ Specification in job description
- ☐ Device availability
- ☐ Own positive attitude
- ☐ Positive attitude and support from own team
- ☐ Positive attitude of the patients
- ☐ Positive attitude of the interprofessional setting
- ☐ Positive attitude from superiors
- ☐ Self-confidence to translate new things into practice
- ☐ Sufficient scientific evidence (e.g. available reference values / knowledge of the state of research)
- ☐ Others: ____

18. Which of the following inhibiting factors (barriers) are relevant to you in relation to the measurement and interpretation of muscle parameter (muscle mass, strength, function)?

Mark all answers that apply.

[multiple response options]

- ☐ Lack of knowledge / understanding of the methods / parameters
- ☐ Lack of practical training / application experience
- ☐ Unclear interprofessional areas of responsibility
- ☐ No willingness of the professional for physical examinations (touch)
- ☐ Poor condition of the patients
- ☐ Circumstances (e.g. insulation)
- ☐ Insufficient organisation / scheduling
- ☐ Missing location / room
- ☐ Insufficient time for application
- ☐ No specification in job description
- ☐ Lack of device availability
- ☐ Negative attitude and lack of support from own team
- ☐ Own negative attitude
- ☐ Negative attitude of the patients
- ☐ Negative attitude of the interprofessional environment
- ☐ Negative attitude from superiors
- ☐ Lack of confidence to transfer new things into practice
- ☐ Lack of scientific evidence (e.g. lack of reference values / knowledge of the state of research)
- ☐ Others: ____

Demographics

A few questions about your professional environment and work experience follow to conclude.

19. What is your professional focus?

Multiple answers are possible

[multiple response options]

- ☐ Malnutrition
- ☐ Adiposity
- ☐ Metabolic disease
- ☐ Cardiovascular disease
- ☐ Diesease of the digestive system
- ☐ Kidney disease
- ☐ Allergies/intolerances
- ☐ Other: _________________

20. In which professional environment do you work primarily?

Please select one answer. If you work in more than one professional environment, select the one with the higher working time percentage.

[only one response option]

- ○ Private practice / freelance — go to question 22
- ○ Hospital (mainly inpatients) — go to question 21
- ○ Hospital (mainly outpatients) — go to question 21
- ○ Rehabilitation clinic — go to question 22
- ○ Nursing home — go to question 22
- ○ Other (research, education, industry, health promotion and prevention) — go to question 22

21. In what type of hospital do you work?

[only one response option]

- ○ University hospital
- ○ Cantonal hospital
- ○ Regional hospital
- ○ Private clinic

22. How many years of professional experience do you have?

[open]

____

23. What is your highest education?

[only one response option]

- ○ In education
- ○ College of higher education
- ○ Bachelor of Science
- ○ Master of Science
- ○ PhD

24. How have you acquired knowledge to collect muscle parameters so far?

Multiple answers are possible.

[multiple response option]

- ☐ Independent literature study / research
- ☐ Webinar
- ☐ Conference / Seminar / Workshop
- ☐ Focused further education (e.g. CAS Nutritional Assessment)
- ☐ During a study program
- ☐ No knowledge acquired

25. Would you be interested in acquiring more information / practical skills on muscle parameters?

[only one response option]

- ○ Yes — go to question 26
- ○ No — go to question 27

26. How would you like to gain knowledge on the measurement/application of muscle parameters?

Multiple answers are possible.

[multiple response option]

- ☐ Articles (e.g. in the SVDE magazine or other journals)
- ☐ Webinar
- ☐ Conference
- ☐ Workshop (1/2 day)
- ☐ Workshop (1 day)
- ☐ Focused further education (e.g. CAS Nutritional Assessment)
- ☐ During a study program
- ☐ Other: ___________________________

27. In which language region do you practice?

[only one response option]

- ○ German
- ○ French
- ○ Italian

28. How old are you?

[open]

____

Closing

29. If you have any suggestions regarding this survey, please feel free to give feedback here:

[open]

____

Closing for participants
Thank you very much for your participation.
You can close the browser window now.

Closing for non-participants
Thank you for your interest.
Unfortunately, you are not part of the target group for this survey.
You can close the browser window now.
