# Supplementary material for: Current Practice of Assessing and Monitoring Muscle Strength, Muscle Mass and Muscle Function during Nutritional Care by Dietitians in Switzerland—An Online Survey
Source: Nutrients. 2022 Apr 22;14(9):1741. doi: 10.3390/nu14091741 (PMC9103861; doi:10.3390/nu14091741)
Supplement: Supplementary file 1 [file nutrients-14-01741-s001.zip › Table S1.pdf]

Table S1: Importance of single parameters for assessment of nutritional status (*n*=117).

**Parameter**	**Response frequency %**					
	Very important	Rather important	Neither	Rather not important	Not at all important	No answer
Body weight	44.4	45.3	7.7	2.6	0.0	0.0
BMI	18.8	47.9	15.4	13.7	4.3	0.0
Upper arm / calf circumference	16.2	47.0	17.9	12.0	1.7	5.1
BIA	42.7	44.4	6.0	1.7	0.9	4.3
DXA, MRI, CT	17.9	27.4	22.2	10.3	4.3	17.9
Handgrip strength	45.3	41.9	2.6	2.6	2.6	5.1
TUG, Chair Stand Test or SPPB	23.1	36.8	12.8	6.0	3.4	17.9
Walking test	17.9	41.0	16.2	5.1	2.6	17.1

Abbreviations: body mass index (BMI), bioimpedance analysis (BIA), Dual-Energy-X-Ray-Absorptiometry (DXA), Magnetic Resonance Imaging (MRI), computed tomography (CT), Timed Up and Go (TUG), Short Physical Performance Battery (SPPB)
