# Supplementary material for: Current Practice of Assessing and Monitoring Muscle Strength, Muscle Mass and Muscle Function during Nutritional Care by Dietitians in Switzerland—An Online Survey
Source: Nutrients. 2022 Apr 22;14(9):1741. doi: 10.3390/nu14091741 (PMC9103861; doi:10.3390/nu14091741)
Supplement: Supplementary file 1 [file nutrients-14-01741-s001.zip › Table S2.pdf]

Table S2: Proportion of consents to statements about the use of muscle parameters (muscle mass, muscle strength, muscle function) (n = 117).

**Statement**	**Consent (%)**
Provides objective data	90.6
Increases the added value of nutritional care	80.3
Adds new scientific evidence to daily clinical practice	78.6
Has a positive effect on interprofessional collaboration	73.5
Improves the relationship between patient and dietician	53.0
Increases professional self-esteem	31.6
Leads to a strong focus on objective parameters	19.7
Leads to demotivation in patients when values get worse	7.7
Worsens interprofessional collaboration if responsibilities are not clarified	7.7
Worsens the quality of counselling through an additional time-consuming assessment	1.7
Other	12.0
