# Supplementary material for: Current Practice of Assessing and Monitoring Muscle Strength, Muscle Mass and Muscle Function during Nutritional Care by Dietitians in Switzerland—An Online Survey
Source: Nutrients. 2022 Apr 22;14(9):1741. doi: 10.3390/nu14091741 (PMC9103861; doi:10.3390/nu14091741)
Supplement: Supplementary file 1 [file nutrients-14-01741-s001.zip › Table S3.pdf]

Table S3: Inclusion of specific muscle parameters in the assessment or monitoring and parameter with the highest considered benefit for monitoring (multiple answers possible).

**Parameter**	**Response frequency %**		
	Inclusion in assessment (*n* = 83)	Inclusion in monitoring (*n* = 87)	Benefit for monitoring (*n* = 117)
BIA	73.5	85.1	82.9
DXA, MRI, CT	1.2	1.1	6.8
Handgrip strength	49.4	50.6	64.1
TUG, Chair Stand Test or SPPB	12.0	11.5	23.9
Walking test	8.4	12.6	25.6
Other	10.8	10.3	6.8

Abbreviations: body mass index (BMI), bioimpedance analysis (BIA), Dual-Energy-X-Ray-Absorptiometry (DXA), Magnetic Resonance Imaging (MRI), computed tomography (CT), Timed Up and Go (TUG), Short Physical Performance Battery (SPPB)
