# Supplementary material for: Current Practice of Assessing and Monitoring Muscle Strength, Muscle Mass and Muscle Function during Nutritional Care by Dietitians in Switzerland—An Online Survey
Source: Nutrients. 2022 Apr 22;14(9):1741. doi: 10.3390/nu14091741 (PMC9103861; doi:10.3390/nu14091741)
Supplement: Supplementary file 1 [file nutrients-14-01741-s001.zip › Table S4.pdf]

Table S4: Cross table language region versus use of muscle parameters for monitoring (n = 117).

Language region	Do you use muscle parameters for monitoring (%)	
	yes	no
German	81.0	19.0
French	55.2	44.8
Italian	75.0	25.0
